# Understanding the Nature of Oneness Experience in Meditators Using Collective Intelligence Methods

**DOI:** 10.3389/fpsyg.2020.02092

**Published:** 2020-09-17

**Authors:** Eric Van Lente, Michael J. Hogan

**Affiliations:** School of Psychology, National University of Ireland, Galway, Ireland

**Keywords:** oneness, non-dual awareness, meditation, collective intelligence, self-perception, space perception, consciousness, well-being

## Abstract

Research on meditation and mindfulness practice has flourished in recent years. While much of this research has focused on well-being outcomes associated with mindfulness practice, less research has focused on how perception of self may change as a result of mindfulness practice, or whether these changes in self-perception may be mechanisms of mindfulness in action. This is somewhat surprising given that mindfulness derives from traditions often described as guiding people to realize and experience the non-separation of self from the world or its “oneness” with the whole of reality. The current study used a collective intelligence methodology, Interactive Management (IM), to explore the nature of oneness experiences. Five IM sessions were conducted with five separate groups of experienced meditators. Participants generated, clarified, and selected oneness self-perceptions they believed most characterized their experience both during meditation and in their everyday experience in the world. Each group also developed structural models describing how highly ranked aspects of oneness self-perceptions are interrelated in a system. Consistent themes and categories of oneness experience appeared across the five IM sessions, with changes in the sense of space (unboundedness), time, identity, wholeness, and flow highlighted as most influential. Results are discussed in light of emerging theory and research on oneness self-perception and non-dual awareness.

## Introduction

The idea that the self is inextricably interrelated to the rest of the world or that everything is part of the same whole can be found in many of the world’s religious, spiritual, and philosophical traditions (Ivanhoe et al., 2018). Examples of this can be seen in Eastern traditions such as Buddhism, Hinduism, Confucianism, and Taoism and in Western traditions such as Christianity and Platonism. These traditions espouse oneness-related concepts such as Nirvikalpa samadhi, Buddha nature, non-dual awareness, Theosis, and Henosis (Taylor and Egeto-Szabo, 2017). Although conceptions of oneness are part of Western culture, they nevertheless present a challenge to more dominant individualistic Western conceptions of a separate self.

Oneness is of interest in part because of the marked life changes that it appears to be associated with. Traditionally, oneness has been considered to be a cause of well-being (Ivanhoe et al., 2018), and evidence of this can be seen in associations between psychological constructs related to oneness and well-being measures. For example, Hood et al. (2009) found mystical experiences to be associated with well-being. Mills et al. (2018) found that non-dual awareness (considered by the authors to be the same as oneness) predicted reduced anxiety and depression. Experiences of oneness may also represent a key mechanism by which mindfulness and meditation lead to increases in well-being. For example, the Nondual Embodiment Thematic Inventory measure has been shown to be a mediator of the effect of meditation practice on anxiety and depression (Mills et al., 2018), and the Metapersonal Self-construal Scale (DeCicco and Stroink, 2007) has been shown to mediate the effect of dispositional mindfulness on well-being (Hanley et al., 2017). Finally, Dambrun et al. (2019) showed that unified consciousness (which included components of self-loss and oneness) mediated the effect of (body scan) meditation on happiness.

Research on oneness dates at least from William James’ (1985/1902) classic text, *The Varieties of Religious Experience*, where he described oneness and its relationship to mystical experiences in the following way:

This overcoming of all the usual barriers between the individual and the Absolute is the great mystic achievement. In mystic states we both become one with the Absolute and we become aware of our oneness. This is the everlasting and triumphant mystical tradition, hardly altered by differences of clime or creed. In Hinduism, in Neoplatonism, in Sufism, in Christian mysticism, in Whitmanism, we find the same recurring note[…] (p. 419)

James suggested four main characteristics of mystical experiences in addition to oneness: (1) ineffability, (2) a noetic quality, (3) transiency, and (4) passivity (a sense in which mystical experiences are involuntary and uncontrollable). Since then, oneness has been considered a core characteristic of mystical or awakening experiences by other researchers. For example, Stace (1960) explicitly included unity in his description of mystical experiences, as well as (2) transcendence of time, (3) deeply felt positive mood, (4) a sense of sacredness, (5) paradoxicality, and (6) persisting positive changes (long-lasting effects in attitude and behavior, resulting from transient mystical experiences). Hood’s mysticism scale (1975) also includes a factor called the “unifying quality,” which relates to oneness. Taylor (2017) noted that oneness – in addition to loss of self-boundaries – is considered to be a “metacharacteristic” of wakefulness, which is considered to cause many other perceptual, affective, conceptual, and behavioral characteristics. More recently, Newberg and Waldman (2017) included a *sense of unity or connectedness* as the first feature of his enlightenment experience categories, in addition to intensity, clarity, surrender (of voluntary control), and sense of permanent change.

In addition to oneness being identified as a core element of mysticism and enlightenment, oneness has also been described as one of the most advanced forms of self-transcendence – that is, changes that involve fundamental transformations in the self or in how the self is perceived. Yaden et al. (2017) define self-transcendent experiences as states that involve dissolved self and self-boundaries combined with connectedness and oneness. This idea also manifests in the context of conceptualizations and measures of meditative pathways to oneness. For example, in the Piron (2001) Meditation Depth Index, a oneness item is included only in the highest level of meditation depth. Similarly, Schoenberg et al. (2018) describe oneness as the ultimate aim of the Buddhist essence of mind techniques rDzogs-Chen [Great Contemplation] and Mahamudra [the “Great Seal” that unites all things]. Mills et al. (2018) describe oneness (non-dual awareness) as a shift in perception of self-awareness, which is the “ultimate aim” (p. 2) of most types of meditation practice, regardless of tradition. Researchers have also developed neuropsychological models involving oneness that make specific predictions about changes in the nature of self-experience.

Three neuropsychological models, the Conscious State Space model (CSS; Berkovich-Ohana and Glicksohn, 2014), the Self-centeredness/Selflessness Happiness Model (SSHM; Dambrun et al., 2019), and the Model of Enlightened/Mystical/Awakened Experience (MEMAE; de Castro, 2017), make predictions about changes in the nature of self-experiences and self-perceptions related to oneness experiences that may result from contemplative practice. All models suggest that meditation may attenuate or diminish the narrative sense of self to a minimal or core self, which is short in temporal extension and exists in the present. The models also differ in certain respects; for example, SSHM and MEMAE also discuss the further attenuation of this remaining minimal self. In CSS and SSHM, state changes can be permanent or trait-like, but this is not assumed to be true in MEMAE, in which oneness comprises only a temporary return to a primal state. Whereas SSHM and MEMAE are three-level self-attenuation models (with effects on well-being predicted only at the third level for SSHM, where both narrative and minimal self-levels are diminished), CSS presumes that changes in well-being and oneness occur as soon at the narrative self is attenuated. While the models require further development and testing, MEMAE posits different neural underpinnings associated with experiences of oneness and non-dual awareness, making it different from most other models that describe oneness and non-dual awareness as cognates. Finally, whereas meditation-induced changes in oneness in SSHM predicted to lead to equanimity and well-being were supported in Dambrun et al. (2019), CCS predictions that contemplative practices should result in lower negative and positive affect trait valences were not fully supported in Berkovich-Ohana and Glicksohn (2017). MEMAE makes no direct predictions about well-being.

The centrality of oneness in many experiences that involve profound changes in the perception of self and the regular consideration of oneness as an advanced form of self-transcendence suggest that developing a construct focused on measuring core features of the experience of oneness could help advance research in the area. One area of research that is central to our current work is the question as to whether oneness experiences mediate the relationship between mindfulness and well-being. However, in order to address this question empirically, a reliable and valid measure of oneness experiences is needed. A number of scholars have developed scales that include items possibly measuring oneness (e.g., see Table 1); however, these scales are not intended to specifically measure oneness experiences. For example, two groups of researchers have developed approaches to measuring *beliefs* in oneness (Garfield et al., 2014; Diebels and Leary, 2019), but not the *experience* of oneness. The Enlightenment Scale (Boyd-Wilson and Walkey, 2015) contains items that resemble “persisting positive changes in attitude and behavior” (Stace, 1960) related to mystical experience but without referring specifically to oneness. Finally, the Nondual Awareness Dimensional Assessment (Hanley et al., 2018) used factor analysis of existing scales, none of which were constructed to selectively and uniquely measure non-dual awareness, but were thought to measure aspects of non-dual awareness.

**TABLE 1:** Sample items that explicitly measure an aspect of oneness from existing scales.

Name of scale	References	Sample oneness item
The Revised Mystical Experiences Questionnaire	Barrett et al., 2015	Experience of oneness or unity with objects and/or persons perceived in your surroundings
Hood Mysticism Scale	Hood, 1975	I have never had an experience in which I felt myself to be absorbed as one with all things
Ego-Dissolution Inventory	Nour et al., 2016	I felt at one with the universe
Nondual Embodiment Thematic Inventory	Mills et al., 2018	Conscious awareness of my non-separation from (essential oneness with) a transcendent reality, source, higher power, spirit, god, etc.
Aspects of Self-Transcendence Scale	Cloninger et al., 1993	I sometimes feel so connected to nature that everything seems to be part of one living organism
Meditation Depth Index	Piron, 2001	I felt myself at one with everything
Oneness Beliefs Scale	Garfield et al., 2014	All existence in the universe forms one great unified life system (belief)
Belief in Oneness Scale	Diebels and Leary, 2019	Beyond surface appearances, everything is fundamentally one./At the most basic level of reality, everything is one./The separation among individual things is an illusion; in reality, everything is one (beliefs)

Drawing upon DeVellis (2017) eight-stage model of scale development, the current study is part of a larger project focused on the development of a scale to measure oneness experiences. The variety of oneness experiences reported in this study was subsequently used to generate scale items for expert review. A limitation of existing oneness-related scales is that they have not been grounded in rigorous qualitative research on a clearly defined experience of a representative group. For example, items for the Enlightenment Scale (Boyd-Wilson and Walkey, 2015) were produced largely based on the first author’s experience and study. Items for the Oneness Beliefs Scale of Garfield et al. (2014) were derived from Ikeda (1982), president of the Soka Gakkai International lay Buddhist organization, and then reviewed and refined by a psychology professor and students. Hood (1975) measure of mystical experience is based on Stace (1960) analysis of texts including expressions of mystical experiences (Hood, 2001), and Hood (1975) work has been critiqued for not independently confirming Stace (1960) categories of mystical experience (van Belzen, 2010). Although there is qualitative work in this area, especially on non-dual awareness (e.g., Paul, 2008; Costeines, 2009; Fire, 2010; McCormick, 2010; Dumetz, 2018), to date statements and categories obtained from this research have not been oriented toward item pool creation for scale development.

The current study sought to understand aspects of oneness experience and how they are interrelated using a consensus-based qualitative methodology. To do so, we used the collective intelligence (CI) methodology, Interactive Management (IM). The IM process is a system of facilitation and problem solving based on John Warfield’s science of generic design (Warfield and Cárdenas, 1993). The current study represents the first application of IM to understanding aspects of oneness experience and interdependencies between oneness experiences. This study first sought to create a comprehensive list of oneness experiences based on the reports of five groups of experienced meditation practitioners. Each group of participants also generated consensus-based models explaining how important aspects of oneness experience they identified are related to one another in an influence structure. The structural models generated across the five CI groups were also combined to generate a high-level meta-analytical structure describing how key categories of oneness experience are interrelated. The results of the study are presented below.

## Materials and Methods

### Participants

Participants were recruited by contacting local meditation and yoga groups and via local media seeking volunteers to join a discussion group about the experience of oneness and non-dual awareness. Male and female English speakers, older than 18 years, who had meditated at least 5 years (1825+ incidences) over their lifetime and who had experienced oneness (“a breakdown of distinction between subject and object”) were invited to participate in the study. Participants’ characteristics are shown in Table 2 below. Because the practitioners in this study are experienced meditators, it was expected that they would report using a variety of meditative practices. However, it was expected that these types of meditative practice would include those covered in previous reviews of meditative practice such as types of mindfulness, mantra, and spiritual meditation (Burke et al., 2017), as well as various types of yoga (e.g., *chitta vritti nirodha*, meaning “the complete settling of the activity of the mind”), which have traditionally strongly overlapped with meditation (Wahbeh et al., 2018) and which are considered an essential component of mindfulness-based stress reduction mindfulness practices (Gallegos et al., 2013). Table 3 reports the range and frequency of different types of meditation practice reported by participants.

**TABLE 2:** Demographic characteristics, meditative experience, of 41 participants across five IM sessions.

Characteristic	%	N	Mean	*SD*
**Age range**				
(Mean, SD)			45.37	9.79
**Gender**				
Male	37	15		
Female	63	26		
**Education**				
Post leaving certificate or some undergraduate	27	11		
Undergraduate	39	16		
Postgraduate	34	14		
**Employment**				
Student	7	3		
Employed/self-employed	83	34		
Other (retired, unemployed, homemaker)	10	4		
**Frequency of meditation (hours on typical day)**				
(Mean, SD)			1.13	0.71
**Years of practice**				
(Mean, SD)			14.8	9.10
**Total hours of practice**				
(Mean, SD)			5,857	5,067

**TABLE 3:** Meditation types most frequently reported by participants.

Meditation type*	No. of times meditators listed this among their four most frequent meditation types**
Yoga	19
Mindfulness	15
Breathing (including yogic)	13
Vipassanâ	9
Sitting	8
Chanting	8
Walking	7
Alexander technique	4
Awareness of awareness	4
Mantra meditation	3
Relaxation	3
Tai chi/qigong	3
Zazen (Shikantza)	3

*Meditation types classified as ‘other’ excluded. **Includes only meditation types that appear 3 or more times.

### Procedure

IM is a facilitated group design process designed to enhance the collective problem-solving ability of groups (cf. Hogan et al., 2015c; Groarke and Hogan, 2016; RezaeiZadeh et al., 2017 for recent social science applications; see Hogan et al., 2014, 2015a,b, 2017 for further details on methodology and application). Four steps were used in the process.

1. The first step involved individual idea generation. Participants were given a description of oneness/non-dual awareness based on definitions provided by Dunne (2011) and Dahl et al. (2015): “Oneness – a term which is often used interchangeably with “non-duality” or “non-dual awareness – has traditionally been considered a state in which a person experiences consciousness in its true form as utterly devoid of subject–object distinctions. It often implies feeling at one with others and the world and a sense that the usual subject–object distinctions are no longer the dominant mode of experience.” Participants were also provided with some additional context. In particular, they were informed that the research team was broadly interested in oneness self-perception (OSP) and how oneness manifests in the areas of thinking, feeling, and action. Participants were then asked to write five ideas based on the following trigger question: “*In what ways do you see yourself as oneness, in terms of your feelings and identity, in terms of how you relate to others, and in terms of your values, goals, and actions?*” Participants were asked to generate ideas based on their own direct experience. These ideas were collated and categorized by the authors in advance of the group coming together for their CI session.
2. The second step involved group idea generation in the CI session, to further advance upon the categorized idea set generated by the group in advance of the session. The current application of IM employed a modified version of the *nominal group technique* for idea generation (NGT; Delbecq et al., 1975). This involved five steps: (i) presentation on wall posters of categorized ideas generated and submitted by the participants in advance of the CI session; (ii) re-presentation of the original stimulus question to participants, “In what ways do you see yourself as oneness…?”; (iii) silent generation of two new ideas in writing by each participant working alone; (iv) presentation of two new ideas by each participant; (v) serial discussion of the listed ideas by participants for the sole purpose of clarifying their meaning and posting of ideas on the idea wall in front of the group, in the category thought to be most appropriate by the participant.
3. The third step involved a closed voting process in which each participant was asked to select five ideas from the idea wall they believed were the most important aspects of OSP.
4. The fourth step involved structuring selected ideas using ISM software. *Interpretive structural modeling* (ISM; Warfield and Cárdenas, 1993) is a computer-assisted methodology that helps a group to identify relationships among ideas and to impose structure on those ideas. This structuring work can be considered an activity in “mapping perceptions” of the group members. The five steps of ISM are as follows: (i) identification and clarification of a list of ideas (e.g., using NGT); (ii) identification and clarification of a “relational question” for exploring relationships among the ideas generated in the previous step. In the current study, given our interest in examining the interdependencies between OSPs, we focused on enhancement relations, specifically, by asking the following question: “*In the context of oneness self-perception, does oneness experience A significantly enhance oneness experience B?*”; (iii) using the relational question to explore connections between pairs of ideas. The group engaged in discussion about each relational question, and a vote was taken to determine the group’s judgment about the relationship. A “yes” vote was entered in the ISM software by the computer operator only if a majority consensus (>70%) was reached; otherwise, a “no” vote was entered; (iv) display and discussion of the structural model based on the group’s judgments; and (v) amendment to the model by the group, if needed.

Overall, the CI sessions each lasted approximately 3 hours. Five IM sessions were conducted. These sessions were facilitated by the lead author.

## Results

Results are reported across three sections. The first section provides an overview of the main categories and themes of OSP generated across the five sessions. The second section describes the five structural models generated during each IM session. The third section presents a meta-analysis of the structural models.

### Category Analysis of Oneness Self-Perceptions

**FIGURE 1:**
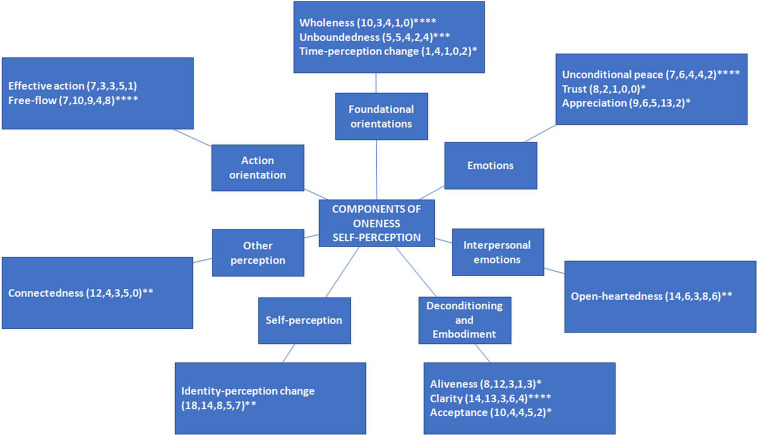
Schematic representation of oneness self-perceptions generated across five IM sessions (number of ideas generated in parentheses across five sessions). Asterisks denote the number of sessions in which statement(s) in this category were selected for inclusion in the structuring phase.

The five sessions generated a total of 377 OSPs. These self-perception ideas were categorized using the paired-comparison method (Warfield and Cárdenas, 1993; RezaeiZadeh et al., 2017; cf. Fauville et al., 2018). This method involves placing eight of the ideas selected at random on a display wall. The next idea in the set is then paired and compared with each of the eight ideas in turn. Ideas conceived as similar to one another are grouped. Groups of similar ideas begin to emerge through repeated cycles of pairwise comparison with new ideas, and as soon as a group includes five similar ideas, it becomes a category that is named by the CI facilitation team. This process is continued until all ideas have been placed into final categories. Using this method, 14 categories were identified, and these categories were further organized into seven higher-order themes (see Figure 1).

**FIGURE 2:**
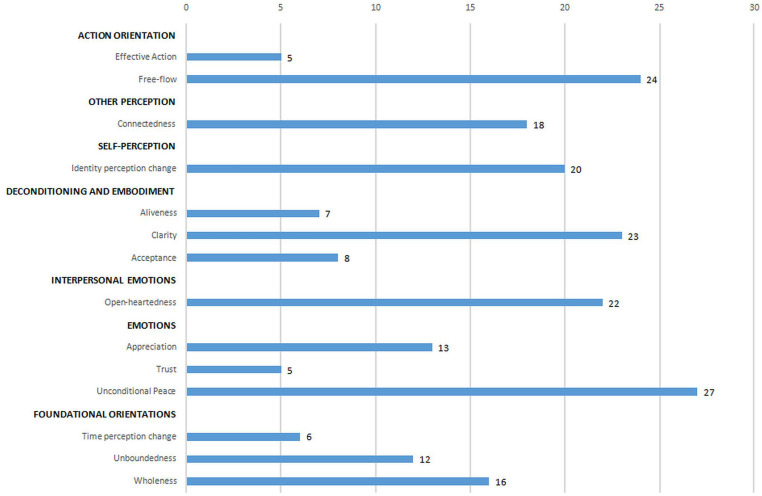
Votes received for each category.

When selecting ideas for inclusion in the ISM structuring phase, participants voted, with patterns of voting reflecting the perceived importance of OSPs. The total number of votes per category is presented in Figure 2.

Table 4 provides a description of each category of experience, along with sample ideas in the category. The first theme, action orientation, includes the categories *effective action* and *free-flow*, and includes a focus on contributing positively, acting in a creative way, and flowing and non-striving. The second theme, which comprised the category *connectedness*, includes a focus on feeling connected to people, nature, and everything. The third theme, self-perception, comprised the category *identity–perception change* and includes ideas that pertain to changes in the sense of self, such as recognizing self as not separate from the world, ego-dissolution, and a sense of commonality beneath differences. The fourth theme, deconditioning and embodiment, includes the categories *aliveness*, *clarity*, and *acceptance*, and includes ideas that pertain to feeling more alive and sensitive, being less attached to thoughts and concepts while being clearer and more insightful, and being less judgmental and more accepting. The fifth theme, interpersonal emotions, comprised the category *open-heartedness* and includes ideas that pertain to being open, understanding, and loving to oneself and others. The sixth theme, emotions, includes the categories of *unconditional peace*, *trust*, and *appreciation* and includes ideas that pertain to a sense of appreciation and joy with fewer negative emotions and a sense of unconditional peace, trust, and support. The seventh theme, foundational orientations, includes the categories of *wholeness*, *unboundedness*, and *time–perception change* and includes ideas that pertain to spaciousness and a sense of boundaries dissolving, to timelessness, to everything being part of a whole and this whole being perfect. The final 2 columns of Table 4 provide descriptions of each category and how participants illustrated ideas in this category.

**TABLE 4:** Themes and categories appearing through this process.

Theme	Category	Clarification	Sample statements from CI sessions, [session number, asterisks (*) are votes]
1 Action Orientation	Effective action	Discerning action, leading to transformative contributions to others and the world	Contributing to society with integrity (4) Actions are usually productive (1)
	Free-flow	A sense of creative freedom and flow which is spontaneous, non-striving and non-resistant	No longer needing to control (4***) Thinking in a way that is fresh and creative (2)
2 Other perception	Connectedness	A feeling of connection with others, nature and fundamental reality, while sensing profound belongingness and familiarity	The experience of being connected with all that is (1*) A feeling of being connected to something greater (2*****)
3 Self-perception	Identity perception change	A sense that one’s self has dissolved, or has shifted away from the normal sense of being a separate thing with a body, thoughts and feelings, often to something less separate	Recognition that nothing can be taken personally (5) Feeling less separate (4)
4 Deconditioning and embodiment	Aliveness	Enhanced aliveness of experience including greater presence, sensitivity, and sensation in the body	A sense of lightness (3**) A sense of presence (1*)
	Clarity	A profound sense of awareness and insight into fundamental reality as well as one’s perceptions, beliefs, actions and their consequences	A recognition that “good” and “bad” depend on thinking (1*) A sense of accessing my true essence (1**)
	Acceptance	A sense of acceptance or non-judgment, of myself, others, and situations	No longer feeling superior or inferior to others (4) A deep acceptance and love for what I am (1*)
5 Interpersonal emotions	Open-heartedness	Being fully open to one’s own and other’s feelings, feeling love and empathy, and treating all people with kindness and compassion	A sharing of myself and my gifts (4*) A feeling of profound empathy (3*)
6 Emotions	Unconditional peace	A stable sense of peace and ease, even in the face of problems, difficulties, and conflicts	No matter what is happening I can sense stillness and peace (1) A focus on peace, regardless of interferences (1*)
	Trust	A sense of unconditional safety, nurturance, and confidence	A sense of trust in inner guidance (1**) A sense of being in a safe haven (2)
	Appreciation	A deep appreciation, valuing and cherishing of life, experienced as bliss, joy, and absence of negativity	A sense of no longer having a fear of death (2) Valuing connecting to inner wisdom (3*)
7 Foundational orientations	Wholeness	A sense that everything – including oneself – is an intrinsic part of a whole and that everything is perfect as it is.	A sense of the world being one energy field (3**) A recognition that suffering is part of oneness (1****)
	Unboundedness	A sense of boundaries dissolving or having dissolved, to reveal unbounded spaciousness and non-separation from everything/anything	A sense of the self-pervading everything (1*) The experience of no distinction between me contained within the body and the world outside (5)
	Time–perception change	A sense that the conventional three-part division of time into past, present, and future no longer fits with one’s experience	A sense of there being no time (2*) The transient nature of experience (5*)

### Structural Models Generated in Each Session

A brief description of the ISM structures generated at each session is presented below. Structures are to be read from left to right, with arrows connecting boxes indicating that the element on the left “significantly enhances” the element on the right. When two or more elements are presented together in the same box, this indicates a reciprocal enhancement relation between elements.

#### Session 1

A total of 130 OSP ideas were generated by participants (*n* = 12) in response to the trigger question. Ideas selected for structuring, along with number of votes received and the associated oneness category, are presented in Table 5.

**TABLE 5:** Session 1 responses to stimulus question: “In what ways do you see yourself as oneness?”

Oneness self-perception category (votes)	Statement
Wholeness (4)	A recognition that suffering is part of oneness
Free-flow (2)	A sense of no longer searching
Trust (2)	A sense of trust in inner guidance
Unboundedness (2)	A sense of being eternal nowness
Clarity (2)	A sense of seeing that our nature is kindness
Clarity (2)	An awareness that all of my actions have an impact on the universe
Clarity (2)	A sense of accessing my true essence

**FIGURE 3:**
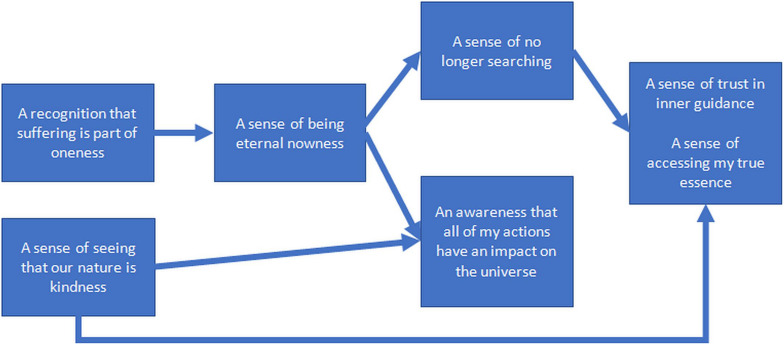
Enhancement structure for the first group of experienced meditators. Structural models generated are to be read from left to right, with relational lines indicating “significantly enhances.” When two or more elements appear together in the same box, this indicates a reciprocal relationship between these elements.

The structural model generated by the group can be seen in Figure 3. Notably, “A recognition that suffering is part of oneness” and “Seeing that our nature is kindness” emerged as the most influential OSPs, in the sense that they are seen to significantly enhance other aspects of oneness. During the group dialogue, it was noted that perceiving oneself as oneness allows one to incorporate suffering in that perception (e.g., as one participant noted: “*In the moment when there’s suffering I’m with it as part of oneness*”). This recognition contributes to a sense of being eternal nowness and an absence of searching. By accepting suffering, both the past- and future-oriented thinking that sustains suffering stops, and there is a perception that time and searching also stop: “*I’m not searching for peace – I’m suffering – that’s it, and it can change but in this moment, I am no longer searching,” and “If there’s nowness…you are not projecting into the future [or] reminiscing about the past, [the] usual triggers for searching…*”

Also, “a sense of being eternal nowness” was judged to enhance “an awareness that all of my actions have an impact on the universe.” It was argued, “*Because if I […] am in the now and I am embodied – well then my actions work and radiate from that, and I am very conscious of that and the outcomes.*” This awareness that all actions have an impact on the universe was also independently seen to be enhanced by a perception “that our nature is kindness.” For example, it was noted, “*you develop a different type of relationship altogether with nature and then you start to see more how the actions of human beings have had a terrible impact on the universe*,” suggesting that a recognition of our nature as kindness also leads to a clearer understanding of the impacts of one’s unkind actions.

This group logic also indicated that “a sense of no longer searching” and “a sense of seeing that our nature is kindness” jointly enhanced the interrelated oneness experiences of “a sense of trust in inner guidance” and “a sense of accessing my true essence.”

With “a sense of no longer searching,” the characteristic motivations associated with searching and striving diminish and trust builds: “*In the no longer searching […] everything is kind of equal – there are no things that are more important than another, no scales of relevance […], equanimous, and within that place you would trust the emergence.” Similarly, one experiences one’s essence: “You are your true essence if you are no longer searching*.”

At the same time, “A sense of seeing that our nature is kindness” was seen as a reason to trust: “*I trust the guidance that I get from that experience,” because “[Kindness] is a trustworthy place*,” and “*with kindness comes trust automatically.” More generally, seeing one’s nature is kindness is simply thought to allow greater trust in it “because you would have connected with it.*”

#### Session 2

Participants (*n* = 12) in this session generated 92 ideas in response to the stimulus question. Ideas selected for structuring, along with number of votes received and the associated oneness category, are presented in Table 6.

**TABLE 6:** Session 2 responses to stimulus question: “In what ways do you see yourself as oneness?”

Oneness self-perception category (votes)	Statement
Unconditional peace (5)	A feeling of inner peace and stillness
Open-heartedness (5)	A feeling of love
Connectedness (5)	A feeling of being connected to something greater
Appreciation (3)	A feeling of great bliss
Wholeness (3)	A sense of being an intrinsic part of all that is
Open-heartedness (3)	Feelings of empathy and compassion
Free-flow (2)	A sense of letting go of all tension in the body
Clarity (2)	A feeling of clarity
Effective action (1)	A sense of complete balance on every level

**FIGURE 4:**
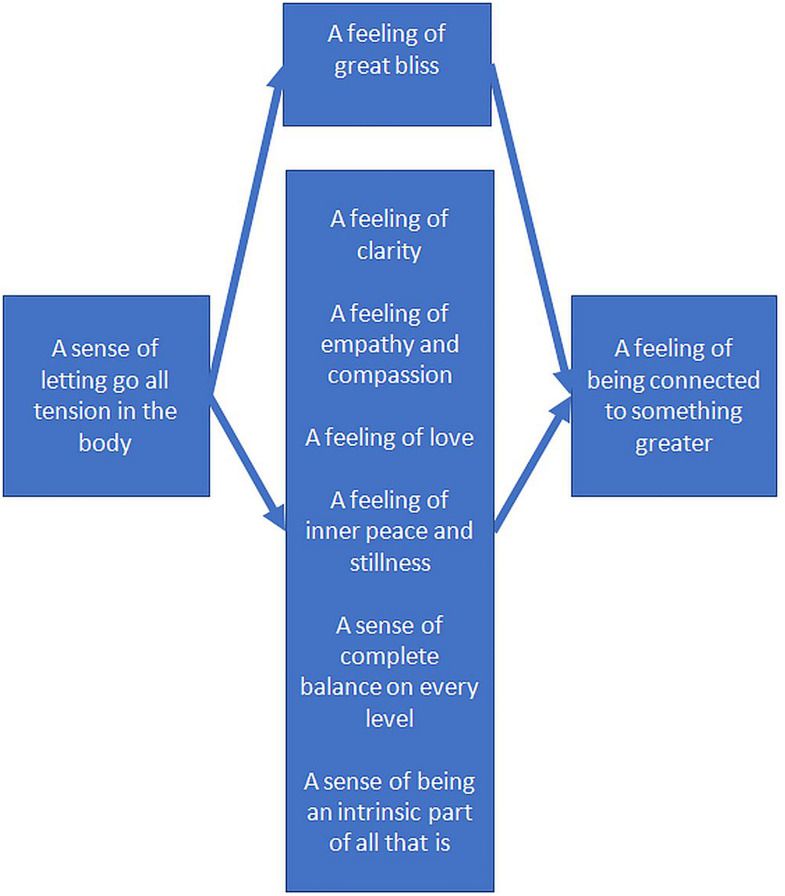
Enhancement structure for the second group of experienced meditators.

This group (see Figure 4) agreed that “A sense of letting go of all tension in the body” influenced all other OSPs, enhancing all elements at level 2 of the structure, including a sense of bliss, and five reciprocally enhancing elements (i.e., feelings of clarity, empathy and compassion, love, inner peace, balance, and being a part of all that is). Participants also judged that letting go of all tension in the body acted through these level 2 elements to indirectly enhance the only level 3 element: “A feeling of being connected to something greater.”

In relation to the reciprocal elements in level 2, it was argued that “feelings of clarity” have a reciprocal enhancement relationship with “feelings of empathy and compassion”: “*If you can have clarity […] if you can step up and look out and without looking at yourself […], you can then see where others or the situation is in – and […] you can truly see it from a compassionate, non-judgmental [place].*” It was also noted that “a feeling of love” and “feelings of inner peace and stillness” reciprocally enhance one another – “*they are just interconnected, each one enhances the other; one doesn’t come first and one doesn’t come second – they are sort of together really.*”

“A feeling of inner peace and stillness” was argued to have a reciprocal relationship with “A sense of complete balance on every level.” It was noted, “*Well I don’t think you can have that balance without the stillness first of all. I don’t think you can have one without the other […], the inner stillness [….] enhances that complete sense of balance at every level.*” Finally, “A sense of complete balance on every level was argued to have a reciprocal relationship with “being an intrinsic part of all that is,” “*Because if you feel a sense of balance on every level, well then you feel you are part of everything else and nature – you are one with everything […] a total part of everything; Ya – [a sense of being an intrinsic part of all that is] can bring about balance.*” Together this group of interrelated oneness experiences was seen to enhance “A feeling of being connected to something greater.” As one participant noted, “*I’d agree with that because for me like – feeling empathic and compassionate is about being open-hearted and if I am open-hearted I feel connected to something greater*.”

At the same time, through a different pathway, “A feeling of great bliss” was seen to enhance “a feeling of being connected to something greater”: “*Well I think that there is a connection between the two because if you feel bliss – you know that something bigger is [happening]. It’s an easy connection to make. Because you experience that … that life force […] and then there’s no self in that experience. So there is no you and there is no object – you are just all energy – you are just part of that energy. Everything is energy.*”

#### Session 3

Participants (*n* = 7) generated 55 OSP ideas in response to the stimulus question. Ideas selected for structuring, along with number of votes received and the associated oneness category, are presented in Table 7.

**TABLE 7:** Session 3 responses to stimulus question: “In what ways do you see yourself as oneness?”

Oneness self-perception category (votes)	Statement
Identity–perception change (4)	A sense of “me” dissolving
Unconditional peace (3)	Feelings of peace and calm
Unboundedness (3)	A sense of being boundless or infinite
Time–perception change (2)	A sense of timelessness
Wholeness (2)	A sense of the world being one energy field
Aliveness (2)	A sense of lightness
Acceptance (2)	A deep sense of acceptance

**FIGURE 5:**
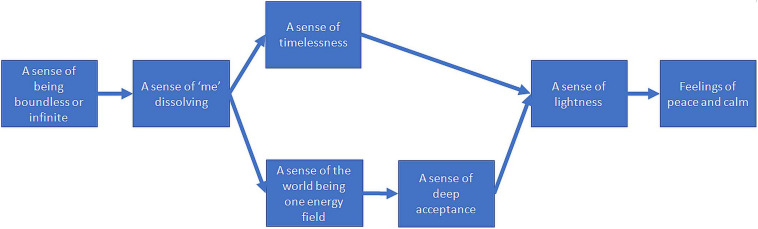
Enhancement structure for the third group of experienced meditators.

The group considered “a sense of being boundless or infinite” to be a critical driver of other aspects of oneness (see Figure 5). While there was some debate as regards the distinction between “A sense of being boundless or infinite” and other ideas (e.g., “A sense of “me” dissolving,” and “A sense of timelessness”), it was seen as broader and more inclusive and enhancing other aspects of OSP: “*it feels like the bigger bubble – the boundlessness* ….”

Participants also argued that “a sense of ‘me’ dissolving” enhances both “a sense of timelessness” and “a sense of the world being one energy field,” which in turn enhanced “a sense of deep acceptance.” The enhancement of “a sense of timelessness” was argued to be mediated through the loss of a “me” that pushed itself to conform to deadlines: “*…if I have less of a […] push with relation to me, if that goes – then there are no deadlines, there’s no time. No need of stuff in the future.*” “A deep sense of acceptance” was explained as a new default state: “*…if there is no me there is nobody to refuse, therefore acceptance is there”;* and “*…If there is no me – you are not there to accept and there is no one there to refuse.*”

There were two pathways of influence to “a sense of lightness,” which in turn enhanced “feelings of peace and calm.” The first pathway is through “a sense of timelessness,” which was seen to produce less heaviness, rigidity, limitation, and hence more lightness: “*if you are conscious of the time then you are bored in your job or whatever. It’s like [a] heaviness and stuckness […], whereas when you are not it’s a lighter sense; at a place when there’s less sense of time there’s also less fixedness*…” Time abundance and less urgency to accomplish and control outcomes, in turn, were seen to produce greater peace and calm: “*There is not major urgency so you are in no rush so you can feel peaceful calmness,” “… you got all the time out there that exists – therefore there is no pressure on your feelings of peace and calm.*”

The second pathway to “feelings of peace and calm” is through “a sense of the world being one energy field” and “a deep sense of acceptance.” Non-acceptance is thought to be possible in a world with one energy field: “*if the world is one energy field therefore there must be acceptance because otherwise there are two: [an] energy field and something else blocking it*.” “A sense of deep acceptance” in turn is argued to lead to “a sense of lightness”: “*once something is accepted then it’s released and there’s no tension there anymore. So it’s like letting go – there’s a lightness in that*,” which enhances “feelings of peace and calm” through relaxing of conflict: “*it’s the lack of acceptance that causes the dissonance and the distortion.*” Finally, the group reasoned that “a sense of lightness” led to “feelings of peace and calm”: “*the feeling of lightness is something that’s quite pleasant normally and not so caught up in everything, so not caught up in the anxiety and the stress that pulls us away from that calm place.*”

#### Session 4

Participants (*n* = 7) generated 59 OSP ideas in response to the stimulus question. Ideas selected for structuring, along with number of votes received and the associated oneness category, are presented in Table 8.

**TABLE 8:** Session 4 responses to stimulus question: “In what ways do you see yourself as oneness?”

Oneness self-perception category (votes)	Statement
Unconditional peace (5)	Feelings of equanimity
Free-flow (3)	A sense of no longer needing to control
Open-heartedness (3)	Treating people with kindness and compassion
Appreciation (2)	A sense that the world is perfect as it is
Connectedness (2)	A sense of belonging
Connectedness (2)	Feeling connected to nature
Clarity (2)	Knowing that I am ultimately not separate from others
Clarity (2)	A sense that the core character of things has been revealed

**FIGURE 6:**
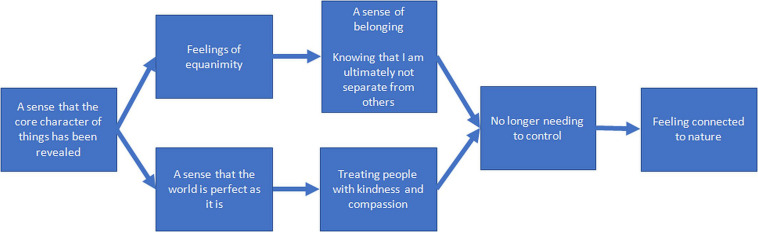
Enhancement structure for the fourth group of experienced meditators.

In the structural model (see Figure 6), “A sense that the core character of things has been revealed” was considered to be a primary driver of all other aspects of OSP. It was argued that this sense that the core character of things has been revealed significantly enhances “Feelings of equanimity,” which in turn enhanced “A deep sense of belonging” and “Knowing that I am ultimately not separate from others.” These aspects of oneness in turn enhanced a feeling of “No longer needing to control.” It was noted, for example, that “*…the need to control – that comes from the fear, if the fear is from the unknown… but if it’s revealed then it’s known”; “[It] breaks down a lot of your fears or doubts… and then it allows you to be more compassionate*.” Furthermore, participants saw equanimity enhancing a sense of belonging because equanimity led to non-separateness: “*[With equanimity] the sense of separateness isn’t there anymore – which leads to a sense of belonging… you realize you belong – everyone belongs.*” Also, in this pathway participants argued that – in their state of not being separated from others they no longer needed to control because they weren’t actually “*running the show […] the* control is sort of not needed […] not really relevant.”

Participants also argued that “A sense that the world is perfect as it is” enhanced a feeling of “No longer needing to control,” because, for example, “… *if it’s perfect* – *then I don’t have to come and put my oar in the way […] and screw it up*.”

“A sense that the world is perfect as it” is also enhanced “Treating people with kindness and compassion.” Participants argued that seeing that things are perfect caused them to treat people with kindness and compassion through being less defensive: “*It’s like letting down the guard – when that comes down – then I’m more available for being kind and compassionate*” and more embracing of what people are like: “*I think you are embracing all of the world and people that are in it – as they are, warts and all – even if you don’t like them and then you treat them kindly and with compassion*.”

In the final part of the structure participants argued that “No longer needing to control” enhanced “Feeling connected to nature,” as with no desire to control nature there resulted in a deeper appreciation of nature and its own way of accomplishing things.

#### Session 5

Forty-one OSP ideas were generated by participants (*n* = 3) in session 5 (see Table 9).

**TABLE 9:** Session 5 responses to stimulus question: “In what ways do you see yourself as oneness?”

Oneness self-perception category (votes)	Statement
Free-flow (3)	A sense of being a conduit rather than a doer
Free-flow (3)	A sense of absence of resistance
Unconditional peace (3)	A sense that experience is suffused with peace even in the midst of great difficulty and conflict
Unboundedness (2)	The experience of dissolution of boundary between inside and outside
Identity–perception change (2)	A recognition of the non-personal yet intimate nature of life
Clarity (2)	A recognition of the futility of striving
	

**FIGURE 7:**
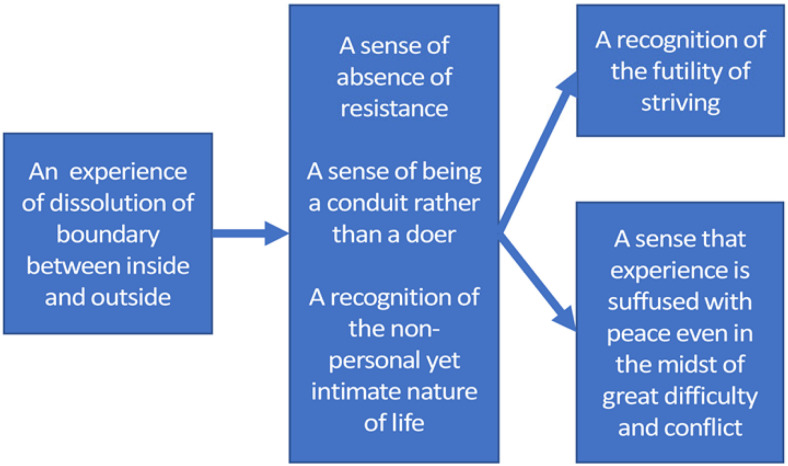
Enhancement structure for the fifth group of experienced meditators.

As can be seen in Figure 7, an experience of “dissolution of boundary between inside and outside” emerged as the most influential component of OSP, influencing three reciprocally interrelated oneness components at level 2 in the structure. Notably, participants argued that “A dissolution of boundary between inside and outside” leads to “A sense of absence of resistance” and enhanced flow (i.e., “A sense of being a conduit rather than a doer”). It was noted, for example, that “it seems that when [the] boundary between inside and outside […] dissolves, there is a kind of transparency […] the sense of me in here and the world out there isn’t there. There’s just a kind of seamless flow of experience.”

At level 2, the reciprocal enhancement was between “a sense of the absence of resistance,” “a sense of being a conduit rather than a doer,” and “a recognition of the non-personal yet intimate nature of life.” For example, it was noted that “*The sense of resistance goes hand in hand with the sense of being a doer. […] There’s a sense of I’m resisting and there’s a kind of self-consciousness – or a doer there*.” The sense of being a conduit rather than a doer was thought to both transcend and include this sense of being a doer: “*…self is a bigger picture than the little framework that is in mind… I can actively live through, and by [non-resisting], mini-me kind of steps out of the way and lets a greater self possess me in the most benevolent use of the word possession.*” The idea of being a conduit rather than a doer also strongly suggested to participants the idea of having no choice to resist – ‘*‘…the conduit just allows through – it’s like […] there is no choice, there is no room to maneuver in terms of making it difficult for myself; “it takes out the sense of resistance already because you are not resisting – you are just the conduit.*”

The set oneness experiences at level 2 in the structure in turn enhanced both “A sense that the world is suffused with peace even in the midst of great difficulty and conflict” and “A recognition of the futility of striving.” In relation to “a recognition of the futility of striving,” it was noted: “*It seems to me that the recognition of the futility of striving is a kind of a seeing – it’s the result of the person seeing*.” Similarly ’A sense that experience is suffused with peace.” was seen to be a result of realizing the non-personal yet intimate nature of life: “*…once you recognize the non-personal intimate nature – […] non-self, then there is this definite experience of peace – whatever is going on*.”

### Meta-Analysis: Influence Map of Oneness Self-Perceptions

Statements from across 13 of the 14 OSP categories appeared in the enhancement structures. A structural meta-analysis of the five models was conducted to understand the relationship between categories of oneness experience. In order to carry out this meta-analysis, the following scores were computed to estimate the influence of each category.

#### Position Score

Each enhancement structure places ideas in stages (Broome, 1995). Ideas to the far right are assigned the lowest position score (i.e., 1), and those in the leftmost stage are assigned the highest score (i.e., depending on the number of levels in the structure).

#### Antecedent and Succedent Score

The antecedent score is the number of elements lying to the left of a particular element (i.e., OSP elements) that enhances it. The succedent score is the number of elements lying to the right of an element in the structure that is enhanced by it.

#### Net Succedent/Antecedent Score

The net succedent/antecedent (Net SA) score is the succedent score minus the antecedent score. If the Net SA score is positive, it means that the specific OSP element is a net source of enhancement. If the Net SA score is negative, it means that the OSP element is a net receiver of enhancement (Broome, 1995).

#### Influence Score

The influence score is the sum of the position score and the net SA score. Influence scores were calculated for each of the 37 OSPs appearing in the five enhancement structures.

**FIGURE 8:**
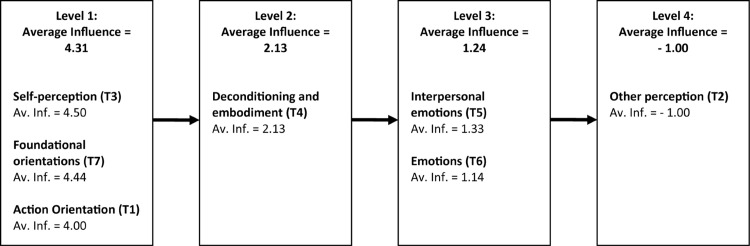
Meta-analysis of influence structures.

Total category influence scores were then calculated by summing the individual element scores, and average category influence scores were then calculated by dividing this summed score by the number of elements in the category. Total theme influence scores were also calculated by summing the individual category scores from across all categories in the theme, and average theme influence scores were then calculated by dividing this summed score by the number of categories in the theme. The meta-analytical model of theme average influence scores is presented in Figure 8. Notably, the categories within the themes “self-perception,” “foundational orientations,” and “action orientation” were considered to exert the greatest influence on other categories of OSP. On the other hand, aspects of oneness experience included in the theme of “other perception” were seen to be influenced by aspects of oneness experience across levels 1, 2, and 3 in the meta-analytical structure.

## Discussion

The current study used a collective intelligence (CI) methodology to identify, rank, categorize, and structure relations between a variety of oneness self-perceptions (OSPs) described by experienced meditators. Participants identified perceptual, affective, cognitive, and activity-related OSPs, some of which had been previously highlighted in religious, spiritual, and mystical experience literature. Notably, a total of 14 categories and seven higher-order themes of OSP were identified. When it came to selecting OSPs for structuring, the top-ranked oneness categories were unconditional peace, free-flow, and clarity. Analysis of the structural models further suggested that the most influential categories of oneness experience were unboundedness, identity–perception change, time–perception change, wholeness, and changes in action orientation. The implications of these results, their relevance to understanding oneness, and implications for theory will be discussed in more detail below.

This exploratory study is the first to use Warfield’s CI methodology to understand OSPs and their interdependencies. Following good practice guidelines, participants were selected based on simple, measurable, and precise inclusion criteria, specifically, declaration of having had a precisely defined oneness experience and a specific minimum meditation experience. Building upon previous exemplars of stakeholder-engaged scale development (cf. Groarke and Hogan, 2016), this study is also the first to employ CI with experienced meditators to understand the oneness construct space and for grounded generation of scale items, which can be used in the development of a oneness experience scale (DeVellis, 2017). The CI methodology involves a consensus-based voting process to select OSPs perceived to be most important and facilitated deliberation in relation to OSP interdependencies, which supports greater understanding and shared reflection in relation to experiences that are often difficult to articulate (Yaden et al., 2016).

Early accounts of mystical and oneness experiences, including James (1902/1985), Stace (1960), and Hood (1975), have conceived of oneness as a special temporary experience restricted to certain contexts and times, perhaps implicitly restricting how it is understood and reported on. On the other hand, the current study asked participants how they experienced themselves as oneness without putting any limits on where, when, or how often they have this experience. Consequently, participants were free to report experiencing oneness not only in the context of meditation or yoga, but also outside of these traditionally meditative contexts. Perhaps partly as a result of this more open-ended approach, participants reported a large diversity of experiences clustered across 14 categories, ranging from changes in the perception of time, space, and self/identity as seen in scales targeted at oneness as self-transcendence and mystical experience (e.g., Hood, 1975; Levenson et al., 2005; Barrett et al., 2015), but also extending into domains of personal and social well-being (e.g., peace, appreciation), cognition (e.g., clarity), and action (e.g., effective action), as seen in other scales more targeted at oneness embodied in everyday life (Boyd-Wilson and Walkey, 2015; Kilrea and Taylor, 2016; Mills et al., 2018). An additional set of statements is familiar and yet reflects an unconditional dynamic that may not be a common everyday life experience (Josipovic, 2016), including *unconditional* peace, well-being, love, and acceptance. The idea of “unconditional” attributes shows similarities to constructs being developed in related research, for example, the idea of serenity (Boyd-Wilson et al., 2004), stable as opposed to fluctuating happiness (Dambrun et al., 2012; Dambrun, 2017) or self-as-context (Moran et al., 2018).

The meta-analysis of influence structures (Figure 8) reveals some patterns that may reflect underlying dynamics in the nature of oneness experience. What most clearly stands out is that the categories of oneness experience associated with changes in fundamental aspects of perception, including space (e.g., “boundlessness”), time (e.g., “timelessness”), self/identity (e.g., “non-separation”), and wholeness (“part of a greater whole”), appear to influence (at level 1) many other OSPs. The fact that these fundamental perceptual categories appear to drive or influence related OSPs is consistent with many descriptions of oneness/non-dual awareness that describe it primarily as a perceptually related shift, for example, in *perception* (Adyashanti, 2009; Mills et al., 2018), *perceptual stance* (Krägeloh, 2019), or *perspective* (de Castro, 2017; Schoenberg and Vago, 2019), Or more specifically a shift in *self-perception* (Brown and Engler, 1980; Mills et al., 2018), *self-perspective* (Hanley et al., 2018), *or identity* (Paul, 2008), for example, where “awareness is viewing the self rather than the self being aware of the experience” (de Castro, 2017, p. 3). More generally, this alteration of time, space, and identity/body is commonly seen in altered states of consciousness research (Tart, 1972; Travis and Pearson, 2000; Shanon, 2003; Vaitl et al., 2005; Hunt, 2007; Ataria and Neria, 2013; de Castro, 2017), with evidence of different neural activity (Berkovich-Ohana et al., 2013; Krause, 2018; Winter et al., 2020; Wittmann, 2020) underlying the experiences of timelessness (outside time) and spacelessness (outside space), related to alterations in the sense of the body and experiences of “then” and “there.” Within contextual behavioral science (Moran et al., 2018), self-as-context is defined as “the coming together […] of a cluster of deictic relations (especially I/Here/Now) that enable observation and description from a perspective or point of view [which] enables or facilitates many different experiences, including […] a transcendent sense of self.”

At the same time, it is also clear that participants see oneness as involving specific affective, cognitive, and interpersonal experiences. One question is if the foundational perceptual changes and changes in action orientation (i.e., free-flow) to the left of the meta-analytical influence structure constitute the core of oneness experience while affective and other changes to the right of these might be “side effects” of these foundational experiences (see, e.g., Adyashanti, 2004). Indeed, this is a debate that has been ongoing in religious schools of thought for some time (e.g., Blackstone, 2012; Josipovic, 2016). While the logic embedded in the meta-analytical structure in the current study does not fully address this question, based on the deliberation of participants across the five CI sessions, the results are at least consistent with the idea that affective and well-being changes may be influenced by perceptual changes, as opposed to the reverse. Moreover, if oneness/non-dual awareness includes well-being as an inherent component (e.g., Josipovic, 2016, 2019), this may present an interesting measurement challenge that has been highlighted earlier (Koenig, 2008; MacDonald, 2017; Visser et al., 2017) – i.e., that a spirituality (or any) measure that is measured using indicators of well-being is correlated with well-being and is therefore tautological. Previous proposals to address this problem are to create pure measures of spirituality (Koenig, 2008) and to permit a maximum percentage (25%) of well-being items in measures (Garssen et al., 2016). An additional solution might be to empirically test models of non-dual awareness including well-being items in a structural equation modeling context and exclude items with cross-loadings across factors. Finally, it is possible that aspects of well-being in the context of non-dual awareness may mean something different to conventional well-being (e.g., because of the unconditional or essentialist nature of some non-dual attributes, such as “unconditional peace”). This may help to mitigate concerns about tautology, once these alternative types of well-being can be accurately measured.

Another finding that comes from the influence structure meta-analysis (Figure 8) points to a possible distinction between two categories of experience that are sometimes considered as equivalent, which is the sense of unboundedness (Foundational Orientations theme) and the experience of connectedness (Other Perception theme). These terms are sometimes used interchangeably (see, e.g., Newberg and Waldman, 2017; Taylor, 2017; Yaden et al., 2017; Carhart-Harris et al., 2018), where unboundedness (or diminished boundaries) is sometimes considered to be an extreme form of connectedness, which is thought to increase along a continuum variously named “sense of connection” (Taylor, 2017), “intensity in perceived self-transcendent unity” (Yaden et al., 2017), and “the unitary continuum” (Newberg and d’Aquili, 2000). In the current study, these categories exist at opposite ends of the meta-analysis influence model and can be interpreted as being qualitatively different. Unboundedness here refers to an absence of boundaries and the closely related experience of the non-separation between apparently different things – such as self and others, or self and the environment, whereas connectedness refers to connection between things while still preserving some separation between these different things. It is not necessarily obvious how connectedness can transform gradually into unboundedness without some additional qualitative change. An alternative view, based on the logic of the meta-analysis influence model, is that oneness involves a qualitative shift in perception that produces a sense of unboundedness rather than this being the presumed endpoint of an incremental growth in perceived connectedness, a presumption that has been called the “presumption of relatedness” (Adyashanti, 2018). This view is also reflected in phenomenological research on non-dual awareness. For example, Dumetz (2018) describes non-dual awareness as a recognition that “no division exists that requires uniting” (p. 14). Costeines (2009) states that “the unity implied by nonduality refers to an already seamless whole rather than the secondary joining together of separate things” (p. 3) and that his participants, “perceive relationships as a process arising within oneness, rather than an exchange between separate people” (p. 181). Direct phenomenological reports that distinguish the experience of connectedness and non-dual awareness include “the deep aliveness of space is so amazing it takes your words away. I don’t feel connected to it. I feel like I am it” (Taylor, 2017, p. 192) and “The sky was me. The trees were me. And so, everything was just ‘me”’ (Lindahl and Britton, 2019). In contrast to de Castro (2017), who describes one possible outward journey of going from everyday filtered (bounded) perception into unfiltered (unbounded) oneness experience, the pathway of enhancement relations across influence structures in the current study point to the return journey as oneness flows from unbounded perception back into everyday life (Wilber, 1999) – influencing everyday emotional, social, and cognitive experiences in the process.

It is also of interest to compare findings of the current research to previous related theory. For example, consistent with the SSHM model (Dambrun et al., 2019) but contrary to the CCS (Berkovich-Ohana and Glicksohn, 2017) hypothesis, well-being is comprehensively represented in the oneness categories and associated statements, including reference to personal, social, and emotional well-being (Keyes, 2002), and a range of character strengths (Peterson and Seligman, 2004). Also consistent with SSHM and CSS models, the influence structure in Figure 8 suggests that changes in the sense of self influence and hence are likely to precede well-being–related experiences.

Partially consistent with SSHM, CSS, and MEMAE models that make predictions about change toward a non-narrative self, the current study points to changes in self that include detachment and disidentification, which suggest a diminishment of the narrative self-phenomenological results, which are also consistent with reduced default-mode activity in neural studies related to meditation, psychedelic use, and mystical experience (Farb et al., 2007, 2016; Garrison et al., 2015; Cristofori et al., 2016; Carhart-Harris et al., 2018).

At the same time, there is little in the current study to suggest that thinking itself is inhibited as part of the oneness experience, apart from one idea (“Being more and thinking less”), as other ideas suggest positive changes in thought content (e.g., creative, spontaneous, clear, and serene thinking, etc.) While some OSPs suggest nowness (e.g., “Being in the present”), a larger proportion of statements generated by participants suggest complete transcendence of time (e.g., timelessness). This suggests that oneness may not entirely exclude experiences (e.g., thinking) associated with the narrative self, but may recontextualize them (Josipovic, 2013).

Strengths of this study include an effort to circumvent biased participant selection by using precise inclusion criteria based on exceeding a minimum required quantity of meditation experience and having had a self-reported experience of non-dual awareness. A related strength of the study is that it responds to calls for an appropriate qualitative approach for use in contemplative studies investigating altered states. In particular, the CI approach is consistent with calls for new methods that “invite phenomenological report and self-interpretation by the participant herself” (Dorjee, 2016, p. 10). The CI approach also supported specific capabilities, including identifying, ranking, categorizing, and structuring OSPs, using a facilitated process to support intersubjective verification of OSPs, while equalizing power and influence of participants, scaffolding of deliberation, questioning and clarification of meaning, selection and ranking of ideas, and consensus-based construction of enhancement structures. Some of these processes have similarities to processes within existing phenomenological approaches such as grounded theory (Garrison et al., 2013) or micro-phenomenology (Petitmengin et al., 2017; Przyrembel and Singer, 2018); however, the collaborative construction of enhancement structures that shed light on the interdependencies between experiences is unique to the CI approach.

### Limitations and Future Research

A number of limitations and directions for future research can be noted. First, the nature of participants’ specific oneness experience was not a direct focus of enquiry in the sense that, other than describing the nature of their experience by reference to component OSPs, which were combined and elaborated upon in CI sessions, we did not measure the extent to which their experience was ongoing or transitory, invoked only during meditation or indeed active in the context of the CI sessions itself, or if it primarily reflected memory of previous experiences or a constructed narrative (Taylor, 2016). This ambiguity could be addressed by more precise participant selection, monitoring, and measurement. For example, some people self-report experiencing oneness in a permanent trait-like way (Costeines, 2009; Goleman and Davidson, 2017), and some people have the capacity to self-invoke oneness-related experiences in a research context (Kelly and Grosso, 2007; Berkovich-Ohana et al., 2013; Dor-Ziderman et al., 2013; Hinterberger et al., 2014; Nair et al., 2017; Schoenberg et al., 2018). These types of participants could be specifically selected.

Second, this study had more female (63%) than male (37%) participants raising possible concerns about generalizability. While this trend is consistent with previous surveys and research suggesting that females (Hood and Francis, 2013) have spiritual experiences more often than males, it is not yet known whether this is also true for oneness experiences specifically. Some limited evidence suggests that women may describe mystical experiences differently than men (e.g., Hood and Hall, 1980; Lange and Thalbourne, 2007), so it is possible that the female-to-male participant ratio may have influenced the data obtained. Future studies could explore possible gender differences in descriptions of oneness experience.

Third, whereas some statements emerging during the CI work were more holistic and appear to bypass the subject–object dichotomy (e.g., “A sense of being boundless or infinite”), other statements indicate subject–object structuring (e.g., “an awareness that all of my actions have an impact on the universe”), and some have a “both/and” quality where things are simultaneously separate and unified (e.g., “A sense of being one with others despite differences”). Although some non-dual traditions hold that language with subject–object structuring could not be describing non-duality (Loy, 1997; Josipovic, 2014), suggesting that participants who employ this language are reporting a recalled experience of oneness from a currently dualistic perspective, alternative explanations are possible. For example, participants may have not yet learned how to articulate their oneness experience using language which avoids subject–object structuring (Proudfoot, 1985); they may be sacrificing the precision of non-dual language structuring for the sake of greater communicability with others who may not yet have had this experience; despite having a persistent background sense of non-dual awareness, dualistic habits of perception may still hold sway in certain areas of their experience (Costeines, 2009, p. 160); they may simply not experience dualistic expression as “not” non-dual and instead experience duality as included within non-duality (Costeines, 2009; Josipovic, 2014), or they may be reporting the dualistically perceived effects of oneness and not oneness itself (see discussion of “connection” above). Recording participants’ current experience while they are reporting about oneness – and probing their individual subject–object language structuring reporting preferences and beliefs – could help distinguish between some of these alternatives.

Fourth, challenges are evident in relation to the definitional starting point of research in this area. Oneness and non-dual awareness have often been treated as equivalent by contemporary spiritual teachers and theorists (e.g., Suzuki, 1962; Nisargadatta, 1973; Wilber, 2001; Maharshi, 2004; de Castro, 2017; Dambrun et al., 2019; Higham, 2019), by qualitative researchers (e.g., Paul, 2008; Costeines, 2009), in experimental interventions (e.g., Mills et al., 2018), and in explorations of underpinning brain mechanisms (e.g., Schoenberg et al., 2018). Where the term “unity” can be considered to have the same meaning as oneness, additional studies also show this equivalent treatment (e.g.,Josipovic, 2014, 2019; Hanley et al., 2018; Schoenberg and Vago, 2019). On the other hand, there has also been a traditional tendency to differentiate non-dual (not-two) awareness from terms such as oneness and unity, which are thought to create dualities simply by having their existence asserted (Loy, 1997). While these disagreements are difficult to resolve, they point to the importance of using increasingly precise definitions of oneness/non-dual awareness in future studies.

Fifth, while this study revealed a diverse range of OSPs, given the use of CI methods across multiple groups, it is possible that a number of different types of oneness are being described, as well as some experiences that precede oneness. For example, there are self-perception changes that involve a sense of non-separation (e.g., “The experience of dissolution of boundary between inside and outside”) and a sense of simultaneous sameness and difference (e.g., “A sense of being one with others despite differences,” echoing Plotinus: “All Being, despite this plurality, is a Unity still.”). At the same time, participants also describe experiences involving disidentification and detachment (e.g., “No longer identifying with objects appearing in awareness”), which paradoxically involves separating from experiences rather than being at one with them. These last two groups of experiences – disidentification and detachment – are called decentering, a process that is hypothesized to be underpinned by experiential selfless processing (Hadash et al., 2016), which involves disidentifying from internal experience. Decentering is often considered to be one of the main aims of mindfulness and related practices (e.g., ACT; cf. Moran et al., 2018), but Hanley et al. (2018) and Dambrun et al. (2019) have also provided empirical evidence that decentering is distinct from oneness. Emerging models of contemplative development (Dorjee, 2016; Schoenberg and Vago, 2019) also suggest that decentering is often a precursor to oneness experiences. On the one hand, findings of different types of oneness and precursors might be considered a strength of this study, particularly given the way in which CI methods allow for interdependencies between different OSPs to be mapped based on the consensus-based deliberations of participants. On the other hand, in terms of coming to understand oneness, the inclusion of decentering-related ideas may suggest that the definition of oneness provided to participants was overly broad. Following from the fourth limitation identified above, this definition could be narrowed in future CI studies.

Finally, the question used to prompt initial idea generation asked participants how they saw themselves as oneness, where oneness was defined both in terms of non-dual awareness (Dunne, 2011; Dahl et al., 2015) and as “feeling at one with others and the world.” To avoid the risk of mischaracterizing this relatively unexplored phenomenon by defining it too distinctly or technically (e.g., using a phrase that many participants may not commonly use or understand), more than one description and phrase were employed in order to allow for the diversity in common descriptions of oneness, as well as its frequent categorization as both an experience (e.g., mystical experience, James, 1985; Yaden et al., 2017) and as a shift in (self-) perception (e.g., Adyashanti, 2009; Leary and Diebels, 2017; Mills et al., 2018) or both (e.g., Paul, 2008). Taking this somewhat broader approach has resulted in respondents providing a number of subtly different consensual (i.e., expressed by five or more participants) descriptions of oneness, consistent with those that have been described in earlier research. These descriptions (available from corresponding author on request) included oneness as “non-separation” (e.g., “No longer feeling separate from anything”), echoing simple definitions of non-dual awareness, but also as wholeness (e.g., “A sense of being an intrinsic part of all that is”), unboundedness/spaciousness (e.g., “A sense of open, infinite space”), recognition of a common unified source (awareness/being) immanent in the diversity of experience (e.g., “A sense that we are all unique and connected via a common source”), and finally as the more common experience of “connectedness” (e.g., “A feeling of connection to everything”). Future CI studies could attempt to better qualify these possible differences in oneness by employing and contrasting single, precise descriptions of oneness or non-dual awareness, and restricting them to refer to either an experience or a change in (self-) perception. While the aforementioned distinction between experience and self-perception change seems likely to overlap with transient/temporary versus trait-based/permanent change, this could also be specifically explored in future research.

## Conclusion

Participants in this study described a range of oneness experiences related to perception, affect, cognition, motivation, action, and interpersonal relations, which are not just relevant to contemplative situations but which also extend to everyday life. These experiences are predominantly positive and include broad well-being effects. Participants also identified enhancement relationships between different oneness experiences, relationships that would be difficult to capture using conventional qualitative methods. This study highlighted the importance of foundational changes in self-perception, space and time, and wholeness together with changes in action orientation (free-flow) as important drivers of other oneness experiences.

The prevalence of reported well-being as part of oneness experiences is broadly consistent with the SSHM model (Dambrun et al., 2019) but differed from the CSS model (Berkovich-Ohana and Glicksohn, 2017). From the perspective of *a priori* theory building, it may be reasonable to assume that well-being is only one of the many broad aspects of oneness experience, and thus not all models would necessarily *a priori* select to include well-being in their definition of oneness. Indeed, the shift in perception commonly associated with this experience is thought to result in a different approach to all of experience – a “non-dual ontology” (Fire, 2010), which may exclude direct reference to emotion or well-being by virtue of the language used in this ontology. Future theory needs to simultaneously explain not only changes in personal and social well-being, but also reported changes in cognition (e.g., an increased sense of clarity and insight, and the “unconditional” and essential nature of some experiences; Josipovic, 2016) and also reported changes in the experience and nature of motivation (e.g., letting go/non-striving).

Moreover, future research should also serve to differentiate between (a) types of oneness, (b) effects of oneness and oneness *per se*, and (c) permanent (e.g., an enduring change in self-perception) and temporary oneness experience (Berkovich-Ohana and Glicksohn, 2017; Taylor, 2017; Dambrun et al., 2019), while also seeking to understand whether oneness is best understood as latent (i.e., “innate”) or learned (de Castro, 2017; Goleman and Davidson, 2017; Josipovic, 2019). Further research can also explore the relationship between decentering and oneness (e.g., Dorjee, 2016) and other potential pathways in the emergence of oneness experiences. Some of these comparisons could be fruitfully explored in future CI studies using more specific context and trigger questions and by contrasting different groups of participants (see Groarke and Hogan, 2016). Other questions are better approached through scale development and structural equation modeling (e.g., Dambrun et al., 2019) and experimental research.

Oneness experience is associated with significant life-changing effects (MacLean et al., 2011; Taylor, 2017). It is also at the core of spirituality (Lomas, 2019). Therefore, understanding oneness experience and its role in well-being is warranted and necessary, something that is made easier as oneness and spirituality are increasingly understood in scientific and secular terms (e.g., Hogan, 2010; Goleman and Davidson, 2017; Lomas, 2019). Greater emphasis on theory building and efforts to understand mechanisms of action in this area may assist researchers and practitioners to design more effective interventions (e.g., Griffiths et al., 2018) that can promote well-being across the lifespan. The current study supports this effort by providing a comprehensive landscaping of oneness experiences, ordered in terms of perceived significance, and structured by reference to perceived interdependencies.

## Data Availability Statement

The datasets generated for this study are available on request to the corresponding author.

## Ethics Statement

The studies involving human participants were reviewed and approved by the National University of Ireland, Galway Research Ethics Committee. The participants provided their written informed consent to participate in this study.

## Author Contributions

MH designed most of the study, reviewed, revised and approved the content of the manuscript. EVL contributed to the design, arranged collective intelligence sessions, carried out most of the data analysis, interpretation of the results, and wrote most of the manuscript. EVL and MH facilitated the collective intelligence sessions. Both authors contributed to the article and approved the submitted version.

## Conflict of Interest

The authors declare that the research was conducted in the absence of any commercial or financial relationships that could be construed as a potential conflict of interest.
